# PVA/Chitosan Thin Films Containing Silver Nanoparticles and Ibuprofen for the Treatment of Periodontal Disease

**DOI:** 10.3390/polym15010004

**Published:** 2022-12-20

**Authors:** Marieta Constantin, Mihail Lupei, Sanda-Maria Bucatariu, Irina Mihaela Pelin, Florica Doroftei, Daniela Luminita Ichim, Oana Maria Daraba, Gheorghe Fundueanu

**Affiliations:** 1 “Petru Poni” Institute of Macromolecular Chemistry, Gr. Ghica Voda Alley 41A, 700487 Iasi, Romania; 2 Faculty of Medical Dentistry, “Apollonia” University of Iasi, 700511 Iasi, Romania

**Keywords:** poly(vinyl alcohol)/chitosan nanocomposite films, silver nanoparticles, ibuprofen, drug delivery systems, periodontitis

## Abstract

Local delivery of drugs or antimicrobial agents is a suitable approach in the management of periodontitis when the infection is localized deep in the pockets and does not adequately respond to mechanical debridement and/or systemic antibiotic treatment. In this context, the objective of this study was to prepare new biocomposite films with antimicrobial, anti-inflammatory, and good mechanical properties to be applied in periodontal pockets. The composite film is eco-friendly synthesized from poly(vinyl alcohol) (PVA) cross-linked with oxidized chitosan (OxCS). Silver nanoparticles (AgNps) were inserted during film synthesis by adding freshly chitosan-capped AgNps colloidal solution to the polymer mixture; the addition of AgNps up to 1.44 wt.% improves the physico-chemical properties of the film. The characterization of the films was performed by FT-IR, atomic mass spectrometry, X-ray spectroscopy, and SEM. The films displayed a high swelling ratio (162%), suitable strength (1.46 MPa), and excellent mucoadhesive properties (0.6 N). Then, ibuprofen (IBF) was incorporated within the best film formulation, and the IBF-loaded PVA/OxCS-Ag films could deliver the drug in a sustained manner up to 72 h. The biocomposite films have good antimicrobial properties against representative pathogens for oral cavities. Moreover, the films are biocompatible, as demonstrated by in vitro tests on HDFa cell lines.

## 1. Introduction

Periodontal diseases represent a health problem with a negative impact on the lifestyle of many people [1]. Periodontitis is a chronic inflammatory disease of the periodontal tissue caused by pathogenic microorganisms and characterized by the destruction of the supporting structures of the teeth [2]. The inflammation is localized in the gum in the early stages, then penetrates deeper and creates bags that will be colonized by anaerobic bacteria that erode the supporting ligaments of the teeth until they are lost [3]. In this context, the prevention of periodontal disease becomes very important, and the best approach is to remove the causes, that is, the formation of bacterial plaque. An effective treatment of periodontitis must control the infection by decreasing the bacterial counts in the affected periodontal pockets, reducing inflammation. Classical treatments, such as the mechanical removal of bacterial plaque and the systemic administration of antibiotics, have confirmed not to be effective enough in the long term, leading the scientific world to find new alternatives [4]. The high drug doses necessary for the systemic and local administration of antibiotics results in undesired side effects owing to the substantial drug concentrations throughout the body. Moreover, bacterial resistances against antibiotic drugs can be developed due to their use for prolonged periods of time. Polymer–drug systems (i.e., hydrogels, gels, films, fibers, micro-/nanoparticles, implants, inserts, etc.) in which the drug is incorporated, encapsulated or adsorbed on the surface have become very promising. The active principle can also be covalently or ionically linked to the polymer chains of the formulation. Moreover, polymer–drug systems are appropriate for local application (external to cutaneous (skin) surface or internal to a mucous membrane, such as eye (conjunctiva), ear, nasal cavity, oropharyngeal cavity, vagina, or anorectal region/tissue for local activity) [5] and can successfully sustain or control the release of antibiotics, antimicrobials, and anti-inflammatory drugs [6]. In practice, one of the major drawbacks of pharmaceutical formulations for local drug administration in periodontitis treatment is the risk of premature, accidental removal from the site of action, due to mechanical stress generated during teeth brushing or chewing. An ideal formulation should remain for a long time at the site of application and release the therapeutic agents in a controlled manner. Compared with other formulations, films are preferred in the preparation of local oral drug release systems because they can be easily prepared and most of the time show flexibility and mucoadhesivity, protecting the inflamed surface area and thus accelerating the healing of periodontal wound. In addition, films can be cut to the desired size and shape so that they perfectly fill the periodontal pocket in which they are inserted [2]. For film preparation, biopolymers, especially polysaccharides and their derivatives, are preferably used as part of the support matrix and for combination with the drug. However, natural polymers do not have appropriate mechanical properties, while synthetic polymers have the disadvantage of a lack of biocompatibility. For these reasons, the combination of the two types of polymers has attracted great interest because the resulting material displays good mechanical properties, easy processability, and low production costs conferred by synthetic polymers, as well as cellular and tissue biocompatibility, characteristic of natural polymers. It must be noted that, currently, devices for the controlled release of drugs made of biodegradable polymer materials are gaining a lot of attention, mainly because after the complete release of the drug, it is no longer necessary to visit the dentist to remove the device.

Chitosan (CS), as a result of its mucoadhesive property and intrinsic antibacterial activity, is preferentially used in the preparation of oral films. This polymer exhibits minimal foreign body reaction, controllable mechanical/biodegradation properties, biocompatibility, hydrophilicity, non-toxicity, non-antigenicity, and antimicrobial activity, as well as bioadherence and cell affinity. Due to its interesting properties, CS is extensively used in biomedical applications as a matrix for drug encapsulation and controlled release [7,8], but also as an antimicrobial agent, alone or with other natural and synthetic polymers [9,10,11,12]. CS is also known to have antimicrobial activity against periodontal pathogens (*Porphyromonas gingivalis* and *Aggregatibacter actinomycetemcomitans*) [13,14] and was used with good results for periodontal treatment in clinical trials [15,16], as well as a film matrix for the periodontal delivery of different drugs, such as tetracycline [17], ciprofloxacin and diclofenac [18], lincomycin [19], metronidazole [20], risedronate [21], metformin [22] or sparfloxacin [23].

Due to their hydrophilic nature, CS-based films cannot work as a barrier to moisture, and for this reason, they were combined with biodegradable synthetic polymers, such as poly(caprolactone) [24,25], poly(vinyl pyrrolidone) [26], poly(lactic acid) [27], poly(lactic-co-glycolic acid) [28], poly(allylamine hydrochloride) [29], and poly(vinyl alcohol) (PVA) [30,31]. Among these, PVA is the most used because it is a non-toxic polymer with a high capability of water absorption, chemical resistance, biodegradability, and biocompatibility properties. Moreover, it has good flexible and foldable film-making properties, all these characteristics making it a suitable polymer for the preparation of flexible membranes for food packages [32,33] and controlled drug delivery systems, including films [34,35] or strip [36] formulations for local application in the periodontal pocket.

The combination of CS and PVA generally has beneficial effects on the biological characteristics of the blend [37]. Furthermore, an additional chemical cross-linking of these polymers improves the mechanical strength and thermal stability, keeping the intrinsic properties of transparency and swelling ability of CS films [38,39]. The most common cross-linking agents involve chemical or ionic interactions among suitable functional groups. The physical cross-linking strategy alone is not efficient enough to obtain mechanically stable films [13,40,41]; therefore, covalent cross-linking procedures have been used. Glutaraldehyde (GA) and genipin are two of the most used chemical cross-linkers for CS and PVA [42,43], known to enhance their mechanical properties. However, GA is considered highly toxic and neurotoxic and genipin is a relatively expensive reagent. In this regard, attempts were made for replacing or combining them with other biocompatible ionic molecules [35]. Dialdehyde chitosan, the oxidated derivative of CS, was successfully used as a cross-linker for chitosan itself or for collagen [44,45,46] by a mechanism almost similar to the low-molecular-weight dialdehyde or dialdehyde starch. It can be expected that materials cross-linked in this way (without additional adding of low-molecular-weight common cross-linkers) will have low toxicity and will maintain chitosan hydrophilicity, an essential characteristic for biomedical applications. Moreover, the cationic nature of CS is important for its interaction with the negatively charged surface of many fungi and bacteria or antibiotics often added to destroy bacteria colonization. As far as we know, this is the first attempt to prepare PVA/CS films by using the oxidized form of CS both as cross-linker and polymeric matrix component.

However, there is no perfect system that can meet all the complex requirements to completely cure periodontitis. New strategies for the treatment of resistant bacterial infections are based on the release of non-antibiotic agents directly at the site of action or the administration of lower doses of drugs. Recent advances in nanotechnology are introducing new therapeutic materials for periodontal regeneration [47]. Silver nanoparticles (AgNps) are one of the most significant metallic nanomaterials with great antimicrobial properties in several types of microorganisms, including oral bacteria [48,49]. They can be applied in the disinfection, prophylaxis, and prevention of infections in the oral cavity. Studies have described the potential of AgNps to combat the dental biofilm reducing the prevalence of dental caries [50], periodontal disease [49,51], and other oral bacterial conditions [49,52]. The incorporation of such agents into a polymeric matrix can increase the wound healing efficacy due to their increased retention at the site of action, sustained release, and greater interaction with the wound environment [53,54,55]. The strategy of capping the AgNps can improve biocompatibility by creating a surface functionalization [56]. Chitosan is a suitable capping agent for the synthesis of metal nanoparticles due to both hydroxyl and amine groups in its structure [57]. The amine group has a strong affinity towards metal ions; hence, it facilitates the binding of chitosan to the metal. Chitosan has been proven to exercise positive effects on the properties of nanoparticles, and thus may contribute to better activities of silver nanoparticles. Moreover, it is expected that CS covering AgNps will not only stabilize the nanoparticles but will also assure the compatibility between the metal and the matrix in the final composite material containing PVA and CS [55,58]. On the other side, even if bacteria are essential agents in periodontal disease, the host’s immune–inflammatory responses play an important role in the destruction of periodontal attachment structures, so non-steroidal anti-inflammatory drugs are also used for periodontitis treatment [59,60]. Ibuprofen (IBF) is a non-steroidal anti-inflammatory drug with analgesic properties, usually orally administered. However, standard oral administration comes with downsides, such as loss of drug availability [61] and gastrointestinal ulcers after prolonged use [62]. Thus, the delivery of IBF through polymer–drug systems is imperatively necessary to avoid these side effects. In this respect, the novelty of this work results from the inclusion in the film of ibuprofen as an anti-inflammatory drug in addition to the silver nanoparticles with antibacterial activity.

**Scheme 1:**
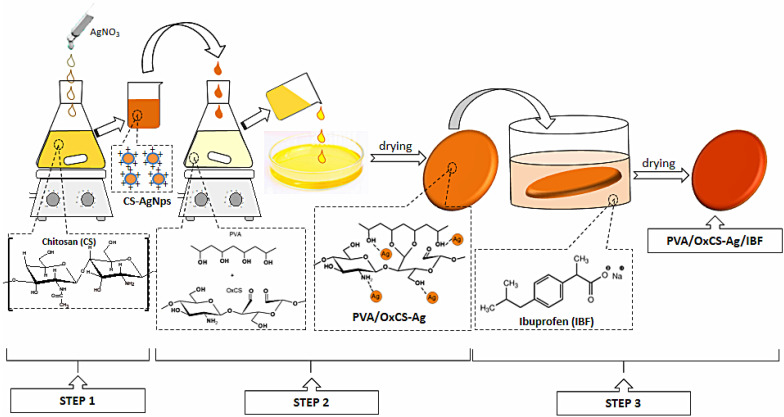
Schematic illustration of the preparation steps for the composite ibuprofen-loaded PVA/OxCS-Ag films.

Considering all this information, the key purpose of this work was to obtain novel composite PVA films using dialdehyde chitosan both as film constituent and biodegradable cross-linker. Firstly, the preparation parameters for AgNps, covered and stabilized by CS, were optimized; then, the composite PVA/CS films were prepared by eco-friendly method, without additional cross-linker, adding the freshly CS-capped AgNps solution into the polymers mixture. A detailed characterization of the films containing different AgNps amounts was realized by scanning electron microscopy (SEM), attenuated total reflection Fourier transform infrared (ATR-FTIR) spectroscopy, and atomic absorption spectroscopy (AAS). Moreover, the mechanical properties, including the adhesiveness of the obtained films, were determined. Ibuprofen was loaded onto the best film formulation, and the effect of drug loading on the film morphology, swelling capacity, and mechanical properties was evaluated. The drug release kinetic was studied, as well as its antibacterial activity against Gram-positive and Gram-negative bacteria. Finally, the cytotoxicity toward human dermal fibroblast adult cells was assessed for all materials in order to choose the best biocomposite PVA/OxCS-Ag/IBF. The preparation steps of the new material are depicted in Scheme 1.

## 2. Materials and Methods

### 2.1. Materials

Chitosan of low molecular weight (CSL); CAS Number 9012-76-4 and medium molecular weight (CSM); CAS Number 9012-76-4, was from Sigma-Aldrich Chem. GmbH, (Taufkirchen, Germany. Poly(vinyl alcohol) (PVA, Mw ~61 kDa, hydrolysis degree 98–98.8%), sodium periodate (NaIO_4_, ≥99.5% (RT)), silver nitrate (AgNO_3_ for analysis), ibuprofen sodium salt (IBF, ≥98% (GC)) were from Sigma-Aldrich Co. (St. Louis, MO, USA) and used without further purification. Acetic acid glacial was purchased from Chemical Company, Iasi, Romania. Distilled water was used as solvent in all experiments.

The viscosity-average molecular weight (Mv) of CSL (193 kDa and the deacetylation degree (DD) of 90%) and CSM (328 kDa, DD of 82%) were determined using the Mark–Houwink–Sakurada equation [63] and ^1^H NMR spectroscopy [64], respectively.

### 2.2. Preparation Methods

#### 2.2.1. Preparation of Chitosan-Capped Silver Nanoparticles (CS-AgNps)

To obtain chitosan-coated AgNps (CS-AgNps), 2 mL of aqueous AgNO_3_ solution of different concentrations was added dropwise over 5 mL of CSL solution in 1% (*v*/*v*) acetic acid. The mixture was stirred at 500 rpm for 30 min at room temperature, then was transferred to an oven and kept for 17 h at the desired temperature. The progression of the reaction was controlled by UV–Vis analysis by using an Evolution 201 UV spectrophotometer (Thermo Fisher Scientific Inc., Madison, WI, USA). The different parameters such as the concentration of AgNO_3_, temperature, reaction time, and concentration of CS solution were optimized.

#### 2.2.2. Preparation of Oxidized Chitosan (OxCS)

Oxidized chitosan (OxCS) was prepared according to the method described by Vold et al. [65]. Chitosan (CSM) (1 g, 6.097 mmol GlcN) was dissolved in 100 mL of acetate buffer pH 4.5 under stirring for 24 h. An aqueous solution (8 mL) of sodium periodate was slowly added (molar ratio IO_4_^−^/GlcN of 4.94/1), and the mixture was maintained for 30 min at room temperature in dark under magnetic stirring. At the reaction time end, an equimolar quantity of ethylene glycol with respect to NaIO_4_ was added, and the stirring continued for another 1 h. Finally, the solution was neutralized with 2.5 M NaOH, transferred to a dialysis bag (Spectra/Por membrane, MWCO = 12,000 Da), and dialyzed against distilled water for 3 days with water change every 24 h.

#### 2.2.3. Preparation of Pure and Composite PVA/OxCS Films

Pure PVA/OxCS films were prepared by mixing a 10% (*w*/*v*) aqueous solution of PVA with the OxCS solution in 2N acetic acid, such as to obtain a gravimetric ratio between the two polymers of 2/1. The pH of the homogenous solution was adjusted at 3 with 1M NaOH, and the mixture (7.5%, *w*/*v*) was kept under slow stirring for 5 h at 60 °C. Finally, the viscous solution was cooled at room temperature, then transferred into petri dishes (Ø = 5.5 cm), and air-dried for 24 h at room temperature and at 40 °C in an oven for another 24 h. After drying, the films were washed for 30 min with 10 mL of 0.5N NaOH, then extensively washed with distilled water to remove the unreacted material and dried at room temperature from ethanol/water mixture (1/1, *v*/*v*).

The composite PVA/OxCS films containing AgNps (PVA/OxCS-Ag) were prepared by the dropwise addition of different volumes of CS-AgNps solution (0.75; 1.5; 2.5; 2.75; and 3.5 mL) to the solution of the two polymers. The gravimetric ratio between PVA and OxCS was 2/1, and the content of CS-AgNps was 1.07; 2.12; 3.15; 4.16; and 6.11% (*w*/*w*) compared with the polymer mixture. Subsequently, the films were prepared and purified similarly to those without nanoparticles.

#### 2.2.4. Preparation of Ibuprofen-Loaded Composite PVA/OxCS-Ag Films

IBF-loaded composite films (PVA/OxCS-Ag/IBF) were prepared by swelling–diffusion method. For this purpose, the neutral –NH_2_ groups were first protonated with HCl 0,1N. Then, 0.10 g dried PVA/OxCS-Ag film was immersed into 20 mL of ibuprofen aqueous solution, 1 mg/mL or 10 mL of 5 mg/mL; molar ratio between NH_2_/COO^−^ groups = 1/1 or 1/2.5). After 48 h, films were washed extensively with distilled water and dried at room temperature. The amount of IBF incorporated was determined spectrophotometrically at λ = 264 nm as the difference between the amount of IBF initially added and that in the washing waters. The quantity of loaded drug in the films (drug loading capacity (*DLC*)) was evaluated by Equation (1):

(1) $$DLC\left(\%\right)=\frac{Amount\;of\;drug\;in\;film}{Amount\;of\;drug-loaded\;film}\times 100$$

### 2.3. Characterization Methods

#### 2.3.1. Determination of the Aldehyde Content of Oxidized Chitosan

The number of aldehyde groups of OxCS was determined by acid–base titration [66] as following: 0.1 g of OxCS and 5 mL of 0.25 M NaOH solution were added to the Erlenmeyer flask and heated at 70 °C on a water bath until the sample was dissolved. After cooling, 7.5 mL of 0.25 M HCl solution and 15 mL of distilled water were added. Then, the sample was titrated with NaOH solution (0.25 M) in the presence of phenolphthalein. The procedure was performed in triplicate for each sample. The content of chitosan units containing dialdehyde groups (oxidation degree (*D.O.*)) was determined using the Equation (2):

(2) $$D.O.\;\left(\%\right)=\frac{C_{1}\;V_{1}-C_{2}V_{2}}{\frac{m}{M}}\times 100$$

where: *C*_1_ (mol/L) and *V*_1_ (L) are the concentration and volume of NaOH solution, and *C*_2_ (mol/L) and *V*_2_ (L) are the concentration and volume of HCl solution, respectively; *m*—mass of the sample (g), and *M*—molecular weight of the repeated unit in dialdehyde chitosan (*M* = 160 g/mol).

#### 2.3.2. Determination of Free Amino Groups by Ninhydrin Assay

The content of free amino groups in CS, OxCS, pure PVA/OxCS films (F #0), and composite PVA/OxCS-Ag films (F #1 ÷ F #5) was determined by ninhydrin assay [67]. The ninhydrin reagent was prepared from 0.8 g ninhydrin and 0.12 g hydrindantin dissolved in 30 mL dimethylsulfoxide (DMSO) and by adding 10 mL of 4 M lithium acetate buffer pH 5.2 while bubbling with nitrogen.

For the assay, to 1 mL of CS and OxCS solutions of different concentrations in 0.5% acetic acid or 1 mg lyophilized film sample in 1 mL 0.5% acetic acid, 1 mL of the ninhydrin reagent was added, and the test tubes were heated at 100 °C in water bath for 30 min. After cooling the solution to room temperature, 0.5 mL was diluted with 10 mL of 50% (*v*/*v*) isopropanol, and the optical absorbance at 570 nm was recorded against a similarly treated blank of 0.5% acetic acid. The concentrations expressed first in mg/mL were converted to molar concentrations (number of moles of amino groups/mL). The concentration of free amino groups in CS and OxCS was then determined from the standard curves of glycine or CS concentrations vs. absorbance. For calculation of the number of free amino groups, a calibration curve of OxCS was used.

#### 2.3.3. Spectral Methods

The *UV–Vis spectroscopic studies* of the CS-AgNps solution and IBF released in the test fluids from composite PVA/OxCS-Ag films were carried out using an Evolution 201 UV spectrophotometer (Thermo Fisher Scientific Inc., Madison, WI, USA).

The *Fourier transform infrared spectroscopy* (FT-IR) of CS-AgNps, pure, unloaded, and IBF-loaded composite PVA/OxCS-Ag films, in their solid (lyophilized) state, was performed on a FT-IR Vertex 70 spectrometer (Bruker, Vienna, Austria) recording in the attenuated total reflection (ATR) configuration, in the range of 4000–600 cm^−1^ at a resolution of 4 cm^−1^. The OPUS 6.5 software was used for the FTIR data processing.

*The amount of silver* in CS-AgNps and composite PVA/OxCS-Ag films was determined using an atomic absorption spectrometer (ContrAA 800 D, Analytik Jena AG, Jena, Germany). The PVA/OxCS films (5–10 mg) loaded with AgNps were digested in 1 mL of 65% nitric acid (HNO_3_) at ambient temperature for 24 h.

*The X-ray diffraction (XRD)* patterns of dried CS-AgNps and CS, PVA, pure PVA/OxCS, and PVA/OxCS films with AgNps were carried out on a Rigaku Miniflex 600 diffractometer (Tokyo, Japan) using CuKα-emission (λ = 1.5406 Å) in the angular range (2θ) of 10° to 90°, at a scanning step of 0.01°, and a recording rate of 5°/min. Background subtraction, smoothing, and WPPF (Whole-Powder-Pattern Fitting) refinement of XRD data were made using the SmartLab II v.4 software package for powder X-ray diffraction analysis, while the diffraction peaks were identified using the Crystallography Open Database (COD).

#### 2.3.4. Dimensional Analysis and Zeta-Potential

The dimensional analysis of CS-AgNps was performed by DLS technique on a ZetasizerNano ZS (Malvern Zetasizer NS-Malvern Instruments, Malvern, UK). The surface charge (ζ-Potential) measurements were also carried out at 25 °C using a Zetasizer Nano ZS at 90° detection angle. The reported data were calculated using the Smoluchowski equation with five separate measurements.

#### 2.3.5. Morphological Analysis

The morphology of CS-AgNps was analyzed by transmission electron microscopy (TEM) using a Hitachi High-Tech HT7700 microscope (Japan) operated in “high contrast” mode and at a 100 kV acceleration potential. Samples were applied from aqueous suspension (1 mg/mL) to 300 mesh copper grids, coated with carbon and dried under vacuum.

The morphology of films (surface and cross-sections) was observed by SEM through a Verios G4 UC Scanning Electron Microscope (Thermo Scientific, Bruno, Czech Republic) equipped with an energy dispersive X-ray spectrometer (EDS, EDAX Octane Elect Super SDD detector, Ametek, Mahwah, NJ, USA). The samples were coated with 10 nm platinum using a Leica EM ACE200 Sputter coater to provide electrical conductivity and to prevent charge buildup during exposure to the electron beam.

The visualization of the surface films (unloaded and IBF-loaded F #3 sample) was carried out by atomic force microscopy (AFM) using Nanoscope IIIa-type Multimode (Digital Instruments, Tonawanda, NY, USA) equipped with an “E”-type scanner. Amplitude- and height-mode images were captured at room temperature in the air using the tapping mode with a silicon tip cantilever (Bruker Corporation, Billerica, MA, USA) operated at a resonance frequency of 275–300 kHz and at a scan rate of 1.2 Hz. The images were evaluated with the Nanoscope V614r1 software (Digital Instruments, Buffalo, NY, USA).

#### 2.3.6. Swelling Studies

The swelling behavior of pure and composite films was determined gravimetrically in phosphate buffer pH 6.8 (66.7 mM, KH_2_PO_4_+Na_2_HPO_4_) at 37 °C. The completely dried, preweighed pure and composite PVA/OxCS-Ag films loaded or not with IBF (~15 mg) were equilibrated in 5 mL of swelling medium for 24 h. At different period times, the films were taken out and blotted with tissue paper to wipe off the surface water, weighed, and immersed again. The swelling ratio (*Q*) was calculated using the Equation (3):

(3) $$Q\;(g/g)=\frac{W_{s}-W_{d}}{W_{d}}\times 100$$

where *W*_s_ is the weight of the swollen film at different time intervals and *W*_d_ is the weight of dried film. Each sample was analyzed in triplicate.

#### 2.3.7. Mechanical Properties

The mechanical properties of pure and composite films PVA/OxCS-Ag and IBF-loaded PVA/OxCS-Ag were investigated using Brookfield Texture PRO CT3(R) texture analyzer (Brookfield Engineering Laboratories Inc., Middleboro, MA, USA). Three wet samples (right after the final washing process) of each type were cut and tested. The films were placed in the measuring holders and subjected to stretching at the speed of 0.5 mm/min at room temperature. The maximum force to break (N) and distance (mm) of probe movement until break was measured, and tensile strength (N/mm^2^) and elongation at break (E%) were calculated. All experiments were conducted at room temperature. The initial length and width of the samples were approximately 30 and 15 mm, respectively. Each sample’s thickness was measured using a digital caliper rule, and the samples were measured in three different points.

#### 2.3.8. Bioadhesive Properties

The strength required to detach the polymeric film from a mucosal surface was applied as a measure of the bioadhesive performance by using Brookfield Texture PRO CT3(R) texture analyzer. A chicken skin mucosa was used as the model membrane, and phosphate buffer solution of pH 6.8 at 37 °C was used as the moistening fluid. The fresh chicken skin mucosa was stored in phosphate buffer pH 6.8 at 4 °C after collection. The lower end of a cylindrical probe (TA5; diameter 10 mm) was glued with a film piece (1 cm^2^), and the cleaned chicken skin mucosa was stuck to the TA-MA fixture kit immersed in phosphate buffer pH 6.8 and thermostated at 37 °C. The skin and the sample were kept in contact for 120 s with 1N force applied during this time. After 120 min, the sample was moved upward (0.5 mm/s) until the complete separation between the surfaces occurred, and the mucoadhesive force was measured with the help of Texture Pro Software. Three films of each formulation were tested, and the average of three determinations was used to estimate the adhesiveness.

#### 2.3.9. In Vitro Drug Release Studies

The drug release properties of the ibuprofen-loaded composite PVA/OxCS-Ag/IBF films (F #3 sample) were studied in phosphate buffer pH 6.8, as the simulated gingival fluid, at 37 °C for 72 h. In order to simulate the application of the film in the periodontal pocket, a static experimental model was adopted in which the film is maintained immovable. Practically, 25 mg of the drug-loaded films (disc with d = 10 mm) were placed in 5 mL of the release medium. Then, 2.5 milliliters of the solution was removed and replaced with the same volume of fresh buffer. The concentration of the drug was determined by UV–Vis spectrophotometer at 264 nm based on a standard calibration curve obtained under the same conditions.

#### 2.3.10. Antimicrobial Activity

The agar disk diffusion method [68] was employed to determine the antimicrobial activity of the synthesized PVA/OxCS-Ag and PVA/OxCS-Ag/IBF composite films against common oral pathogens. Gram-positive bacteria such as *Staphylococcus aureus* ATCC 25,923, and Gram-negative bacteria such as *Pseudomonas aeruginosa* ATCC 27,853, *Klebsiella pneumoniae* ATCC BAA-1705, and *Porphyromonas gingivalis* ATCC 33,377, were grown into broth culture for antimicrobial assay. For disk diffusion test, suspension of each strain of 0.5 McFarland density was inoculated on a plate with the substrate (Chapman Agar for *S. aureus*, MacConkey Agar for *K. pneumoniae*, Pseudomonas Cetrimide Agar for *P. aeruginosa*, and Columbia Agar with 5% Sheep Blood for *P. gingivalis*) (Thermo Fisher Scientific, MA, USA). Film disks with 10 mm diameter were carefully placed on petri dishes and incubated at 37 °C for 24 h. The diameter of the zone of inhibition was measured in millimeters and pure PVA/OxCS film was used as a negative control. Three parallel samples were adopted for each experiment.

#### 2.3.11. Cytocompatibility Assay

Cytotoxicity evaluation of pure and composite films, PVA/OxCS-Ag and PVA/OxCS-Ag/IBF, was examined using MTT assay per standard protocol. Approximately 10^4^ HDFa (human dermal fibroblast adult) cells were seeded in a flat-bottomed 96-well polystyrene-coated plate and were grown for 24 h at 37 °C in a 5% CO_2_ incubator. Film disks of 4 mm diameter (~2 mg) were UV sterilized for 5 min prior to the incubation in the presence of cells. For comparison, 2.5 mg/mL stock solutions of IBF were prepared in sterile distilled water. Series of dilutions equivalent to 600 μg/mL, 400 μg/mL, 300 μg/mL, 200 μg/mL, 150 μg/mL, 100 μg/mL or 50 μg/mL of IBF in the medium were added to the plate and incubated for 6, 24 or 48 h. Cells without drug or film samples were used as control. To determine the cell viability, at the end incubation time, MTT dye (10 μL from 5 mg/mL stock) was added to each well and incubated for 4 h at 37 °C with 5% CO_2_ in dark. The formazan crystals formed by cellular reduction in MTT were dissolved in DMSO (100 μL) solution and incubated for 10 min at 37 °C. The absorption was measured at 570 nm by using a Multiskan FC reader (Biotek, Germany). The percentage of viable cells was calculated according to Equation (4):

(4) $$Cell\;viability\;\left(\%\right)=\frac{Sample\;absorbance}{Control\;absorbance}\times 100$$

The average values of 3 independent experiments (each time all conditions were repeated with triplicate wells) were considered.

### 2.4. Statistical Analysis

All experiments including biological tests were performed in triplicate, and the experimental values are reported in the form of average ± standard deviation. Results were statistically compared by applying a multifactorial analysis of variance (ANOVA) with *p* < 0.05 using GraphPad Prism 6 software.

## 3. Results and Discussions

### 3.1. Synthesis and Characterization of CS-AgNps

It is well-known that AgNps absorbs radiation in the visible region of the electromagnetic spectrum (about 380 to 450 nm) due to the excitation of surface plasmonic vibrations, which are responsible for the color of different intensities of AgNps from yellow to brown. In this respect, the UV–Vis spectrum of AgNps synthesized in the presence of CS were recorded and used as a reference for discussing their optical properties.

**Figure 1:**
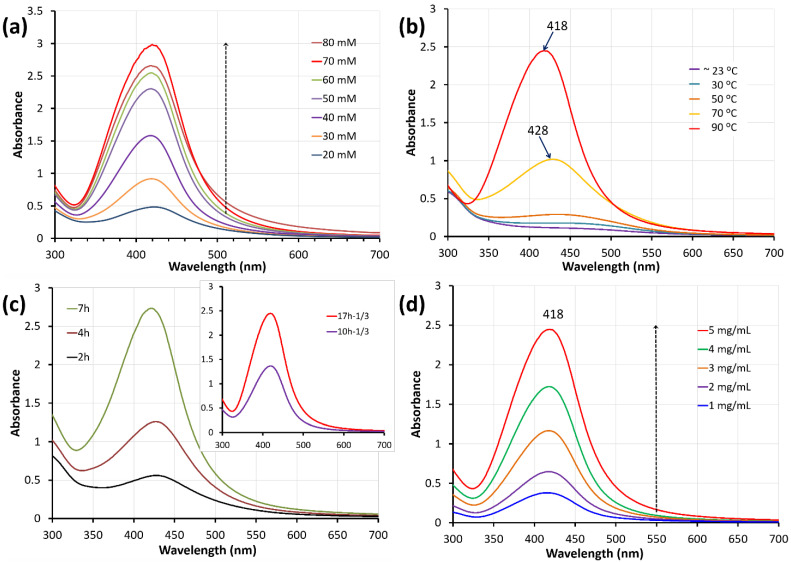
UV–Vis spectra of synthesized CS-capped AgNps in different conditions: (**a**) different AgNO_3_ concentrations ((CS) = 5 mg/mL; 90 °C; 17h); (**b**) different temperatures ((CS) = 5 mg/mL; (AgNO_3_) = 70 mM; 17h); (**c**) different reaction times ((CS) = 5 mg/mL; (AgNO_3_) = 70 mM; 90 °C); (**d**) different CS concentrations ((AgNO_3_) = 70 mM; 90 °C; 17h).

Figure 1 shows the UV–Vis spectra of synthesized CS-AgNps obtained after modification of different parameters. A narrow intense peak of different heights around 420 nm was observed for nanoparticles synthesized using 5 mg/mL CS in 1% acetic acid (17 h at 90 °C) by varying AgNO_3_ concentration (20 ÷ 100 mM) (Figure 1a). The occurrence of a peak at this wavelength (λ_max_ value) reflects the size of AgNps around 30–40 nm [69]. As the concentration of AgNO_3_ increases from 20 to 70 mM, an increase in the maximum absorption is observed, while with a further increase, the intensity of the absorption decreases, which suggests an agglomeration process of the nanoparticles. All preparations maintained at 23, 30, and 50 °C showed poor yield of biosynthesis (Figure 1b). The high intensity and the shift of the maximum absorption at 418 nm, as well as the symmetrical shape of the absorption peak of the CS-AgNps synthesized at 90 °C, are an indication of the formation of Nps with small and uniform sizes. The concentration of CS-AgNps obtained at 90 °C increased greatly, the absorption spectrum in Figure 1b being recorded by the threefold dilution of the initial solution. Moreover, an increase in the intensity of the absorption spectrum, which becomes narrower and symmetrical, with increasing reaction time was observed (Figure 1c). All absorption spectra of CS-AgNps obtained in reaction mixtures of 70 mM AgNO_3_ with varying concentration of CS solution (1 ÷ 5 mg/mL) (Figure 1d) correspond to 3/1 dilutions of the initial solutions, proving that a large number of nanoparticles are obtained. The absorbance of the peaks increases by increasing the CS concentration, the maximum absorption moving from 425 to 418 nm. The absorption spectrum narrows and becomes symmetrical at higher polymer concentrations, indicating a uniform distribution and a small size of AgNps. On the chosen concentration range, there is no tendency to agglomerate AgNps, CS having a role both as a reducer of silver ions and as a good stabilizer of the obtained nanoparticles. Optimal conditions for the synthesis reaction of CS-AgNps were found to be 5 mg/mL CS solution, 70 mM AgNO_3_, 90 °C, and 17 h.

**Figure 2:**
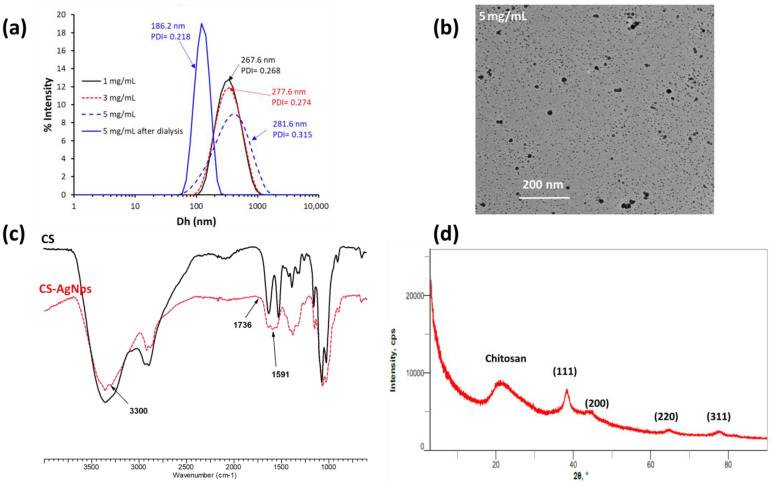
Size distribution (measured by DLS) of CS-AgNps synthesized using different CS concentrations (**a**) and TEM images (**b**); FT-IR spectra (**c**) and XRD spectrum (**d**) of CS-AgNps (experimental conditions: (CS) = 5 mg/mL, (AgNO_3_) = 70 mM; 90 °C; 17 h).

The dimensional distribution of AgNps in aqueous solution, measured by DLS right after preparation, was monomodal, and the average size was 281.6 ± 12.06 nm (Figure 2a), slightly larger than those obtained after purifying by dialysis (186.2 ± 9.75 nm). The polydispersity index (PDI) of 0.218 indicates a monodispersed pattern of nanoparticles [70], and the Zeta potential analysis (ζ = 26.6 mV) also suggests that these AgNps are stable in nature. TEM images (Figure 2b) of AgNps obtained using CS concentration of 5 mg/mL confirmed that they have a round shape and sizes between 7 and 10 nm in dried state.

Evidence of the reducing and stabilizing role of CS was verified by FT-IR and X-ray spectrometry (Figure 2c,d). The FT-IR spectrum of CS-AgNps (Figure 2c) shows specific absorption bands for the stretching vibrations of the N-H bond at 3000–3360 cm^−1^ [71]. The significant decrease in the intensity of this band indicates that the vibration of the N-H bond was affected by the attachment of silver [72]. In addition, the reduction in the intensity of bands at 1635, 1591, and 1558 cm^−1^ corresponding to the C=O stretching of amide I, N-H bending of the primary amine [73], and amide II, suggests the attachment of silver to nitrogen atoms, because the molecular weight becomes higher after silver binding [74]. A new small peak can be observed at 1736 cm^−1,^ which can be attributed to carbonyl stretch vibrations in ketones, aldehydes, and carboxylic acids and may indicate a concomitant oxidation of hydroxyl groups in CS and/or its hydrolysates [75]. The XRD diagram in the 3–90° region for CS-AgNps after dialysis and drying by lyophilization is shown in Figure 2d. It can be seen that all the diffraction peaks corresponding to the reflections in the planes (111), (200), (220), and (311) at 2θ = 38.23°, 44.37°, 64.82°, and 77.64° appear in the spectrum. Indeed, the positions of the peaks and the width of the Bragg reflections are specific to silver with a face-centered cube crystal lattice, indicating the formation of metallic silver (in the zero-valence state) (JCPDS file No 01-071-4613). The peak that appears at 2θ = 22.28° belongs to chitosan according to the literature data [74,76].

### 3.2. Synthesis and Characterization of Oxidized Chitosan (OxCS)

**Figure 3:**
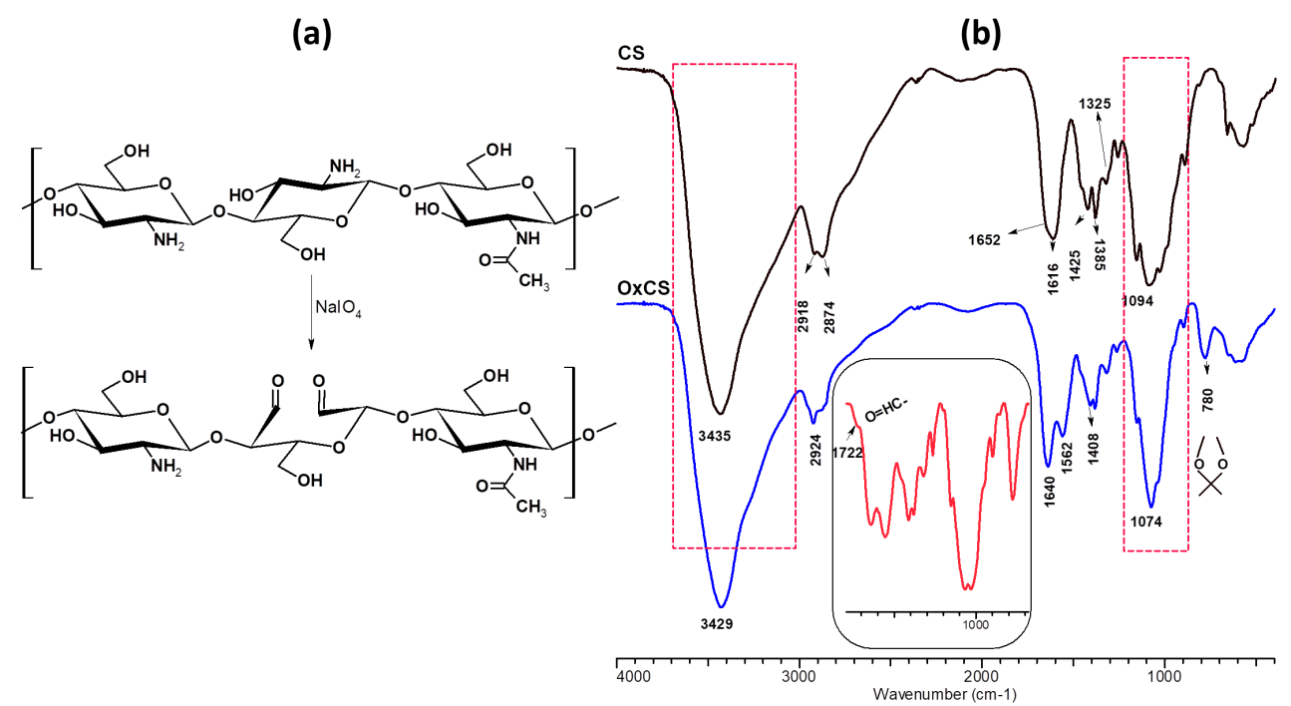
Schematic representation of CS oxidation (**a**) and FT-IR/ATR spectra of CS and OxCS (**b**).

Chitosan with Mv of 32.82 × 10^4^ g/moL was modified by an oxidation reaction using NaIO_4_ (Figure 3a) in order to introduce dialdehyde groups on the polymer chains. The dialdehyde derivative (OxCS) is able to play a double role, namely film matrix component and cross-linking agent. The periodate oxidation reaction is known to produce a depolymerization of the polysaccharide chain as a side effect [77]. Therefore, using a IO_4_^−^/UGU molar ratio of 1/4.94, we obtained a CS with a degree of oxidation of 30.1% and a molecular mass of 8.32 × 10^4^ g/moL. The ninhydrin test indicated a 15,8% loss in the amine content between the native CS (1.9 mequiv./g) to its oxidized form (1.6 mequiv./g) due to the loss of the amine groups during the periodate oxidation reaction.

In the Figure 3b is depicted the FT-IR spectrum of the OxCS compared with that of pure powder CS. In the spectrum of pure CS, the carbonyl band of the amide I was observed at 1652 cm^−1^ and the NH_2_ band at 1616 cm^−1^. At 3429 cm^−1,^ OxCS showed the stretching band of the OH and NH groups, the C-H stretching bands at 2924 and 2874 cm^−1^, and the characteristic bands of amide I (O=C-NHR) at 1640 cm^−1^, as well as amine and the symmetric deformation of NH_3_ groups at 1562 cm^−1^ [78,79]. The change in region 1000 to 1200 cm^−1^ corresponds to the stretching vibrations of the C-O-C bonds in the polysaccharide backbone [78]. The new band at 780 cm^−1^ in OxCS can be attributed to hemiacetals formed by the interaction between aldehydes and alcohol groups, while the shift in the wavenumber region of 1550–1650 cm^−1^ can be related to some interaction between acetic acid and the nitrogen donors of the CS [79] or to the formation of imine bonds between aldehyde and amino groups (Schiff bases) [44]. The typical signal of aldehydic carbonyl groups around 1720 cm^−1^ was detected as a small shoulder only in the ATR spectrum of OxCS (Figure 3b, inset), since the aldehydes interact with neighboring alcohol and amino groups [80]. 

### 3.3. Formulation and Characterization of PVA/OxCS Films without and with AgNps

In order to prepare film samples with good mechanical properties (provided by PVA) and sufficient ionic groups (-NH_2_, given by CS) for drug incorporation by electrostatic interactions, the cross-linking agent (OxCS) was used in high percentage (50% relative to the amount of PVA) (Table 1). In fact, for the next experiments, the best film formulation with adequate properties such as hydrophilicity, strength, adhesiveness, and uniform distribution of silver nanoparticles is desirable.

#### 3.3.1. Chemical Structure

**Figure 4:**
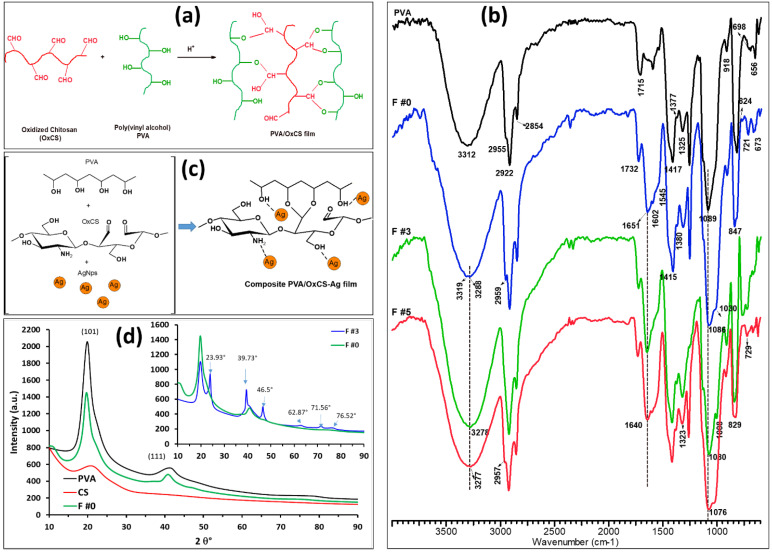
Schematic representation of the synthesis mechanism of pure (**a**) and composite PVA/OxCS-Ag films (**c**); FT-IR/ATR (**b**) and XRD (**d**) spectra of pure (F #0) and composite PVA/OxCS-Ag films (F #3 and F #5).

The pure PVA/OxCS and composite PVA/OxCS-Ag films were prepared by solvent casting method via acetal linkage between the OH groups of PVA and aldehyde groups of OxCS (Figure 4a) (at Ph = 3 and 60 °C). The evidence of the cross-linking reaction results from FT-IR analysis (Figure 4b). Thus, the FT-IR/ATR spectra of the pure PVA film consist of a strong broad peak centered at 3312 cm^−1^, which is linked to the stretching vibration of O-H from the intermolecular and intramolecular hydrogen bonds [81]. The bands between 2800 and 2960 cm^−1^ correspond to asymmetric and symmetric stretching vibration of the saturated C–H bonds, while the peak at 1715 cm^−1^ is from the carbonyl group of the remaining non-hydrolyzed vinyl acetate group of PVA [82,83]. Two peaks at 1659 and 1600 cm^−1^ may be associated with conjugated diones or single carbonyls in a conjugation with C=C double bonds in a solid state [84]. The bands at 1417 and 1325 cm^−1^ are related to the secondary O-H in plane bending and C-H wagging vibrations, and the peak at 1090 cm^−1^ corresponds to C–O stretching vibration of PVA [85]. The FT-IR spectrum of F #0 films (neutralized with NaOH) showed new absorption peaks corresponding to the amide I (1651 cm^−1^), free –NH_2_ bending at 1602 cm^−1^ and amide II (1545 cm^−1^) from OxCS, attesting its presence in film (Figure 4b). The stretching vibration of OH and NH groups from PVA and OxCS appeared at 3306 cm^−1^ and the band at 1089 cm^−1^ corresponds to the glycosidic linkage. A new peak appeared at 1030 cm^−1,^ which can be assigned to the O-C-O acetal group vibrations alike to the glutaraldehyde cross-linking reaction of PVA [44,86].

The composite PVA/OxCS-Ag films were prepared by using a fresh CS-AgNps solution (2.18 mg/L Ag, as determined by AAS), without purification (Figure 4c). Thus, five composite film samples that differed in AgNps content were prepared (Table 1).

The chemical composition of PVA/OxCS-Ag-based composite films determined by AAS showed a slight difference between the theoretical and practical content in Ag; this difference being greater when larger amounts of CS-AgNps were used (F #4 and F #5 samples in Table 1). Moreover, the final content of CS and PVA in the films changed when larger amounts of CS-AgNps were added. In fact, the presence of AgNps hinders to some extent the cross-linking of the two polymers. As a result, free PVA leaves the polymer mixture during the washing step. However, quite large amounts of AgNps could be incorporated by this method, but the use of larger amounts of AgNps (>4.16 wt.%) leads to their agglomeration. Similar results were reported by Popescu and co-workers [58] and Cadinoiu and co-workers [55], who prepared composite CS/PVA hydrogels or films by using preformed CS-capped AgNps. FT-IR/ATR spectra (Figure 4b) of the composite films containing AgNps show all the characteristic peaks mentioned above for pure PVA/OxCS film (F #0); the similarity in the spectra representing a sign of the proper dispersion of filler materials in the film matrix [83]. However, there are slight shifts in peaks of PVA/OxCS-Ag films (F #3 and F #5 samples), such as at 3300 cm^−1^ (stretching vibration of OH/NH_2_ groups in PVA and CS) to 3278 and 3276 cm^−1^, 2959 cm^−1^ (CH_2_ asymmetric stretching of PVA/CS) to 2957 cm^−1^, 1651 cm^−1^ (amide I band) to 1640 cm^−1^, 1325 cm^−1^ (C-H wagging vibrations in PVA) to 1323 cm^−1^, and 1086 cm^−1^ (C-O stretching in PVA/OxCS) to 1080 and 1076 cm^−1,^ indicating that the AgNps are H-bounded to the functional groups present both in CS and PVA. The intensity of these bands increased in the F #3 sample due to stretching of the polymeric backbone chain by AgNps incorporation, resulting in the increasing hydrophilicity of the composite film [83]. Oppositely, a decrease in the intensity of the peaks observed for the F #5 sample indicates stronger H-interaction between AgNps and the polymeric film matrix, with an increase in the hydrophobicity as a result of the presence of metallic nanoparticles. Additionally, new peaks were observed in the composite films in the region 600–1000 cm^−1^ that may confirm the presence of Ag nanoparticles [87].

The X-ray diffraction (XRD) spectra of CS, PVA, and pure PVA/OxCS films (F #0 sample) are shown in Figure 4d. The presence of two distinct diffraction peaks at 19.68° and 40.85° can be observed, which are due to the (101) and (111) planes of the monoclinic unit cell [88], respectively. These peaks are also seen in the XRD spectrum of PVA film. However, the intensity of the two peaks decreased in the F #0 film, suggesting the presence of a more amorphous structure resulted after cross-linking with OxCS. In fact, the structure change can be a consequence of the acetal bond formation, which weakens the intermolecular H-bonding responsible for the semicrystalline nature of PVA. Broad XRD patterns of CS film at 22.28° indicate its semi-crystalline nature. The diffractogram of the composite PVA/OxCS-Ag films (F #3) (Figure 4d, inset) showed six peaks at 2θ values assigned to (113), (101), (231), (110), (103), and (112) planes of silver cubic crystalline structure [89], which is closely connected with the JCPDS (Card No. 04–0783) for silver [90]. The diffractogram pattern confirmed the presence of random-oriented metallic silver nanoparticles in the PVA/OxCS-Ag films.

#### 3.3.2. Morphological Characterization

**Figure 5:**
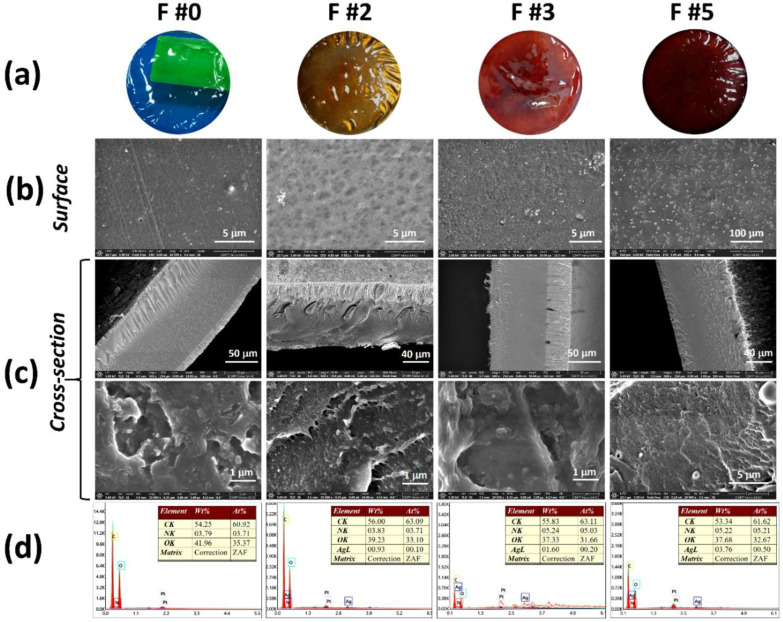
Optical images (**a**), SEM microphotographs of surface (**b**) and cross-section (**c**), and Energy-dispersive X-ray spectroscopy (EDX) spectra (**d**) of PVA/OxCS and composite PVA/OxCS-Ag films.

The PVA/OxCS film of ~0.3 mm ± 0.02 thickness in dried state was soft and transparent (Figure 5a). The incorporation of metallic nanoparticles in PVA/OxCS films was evidenced by progressive coloration of the films. SEM images of pure and AgNps-loaded PVA/OxCS films are presented in Figure 5b. The surface of the film without AgNps (F #0) is flat, relatively smooth, without any pores or cracks, and with small particles very sparsely distributed without any phase separations. Similar to other CS/PVA matrix, it could be detected that CS microdomains dispersed in the film with relatively good interfacial adhesion between the two components [91]. An aggregation of AgNps on the surface was evident for the films loaded with higher amounts of AgNps (F #5). The results are similar to those previously reported on CS/gelatin/AgNps [11] or CS/PVA/AgNps hydrogels [58] and films [83]. Analysis of the cross-section images indicated that the films are homogeneous in mass confirming that the cross-linking process takes place in the entire volume of material. The F #2 sample displayed a slightly porous surface, which is in agreement with the FT-IR results, suggesting an increase in the hydrophilicity in this sample. The presence of AgNps (white spots) in the cross-section of the composite films was evidenced only for the F #5 sample and confirmed their homogenous distribution within the structure of films. However, EDX results from the cross-section of composite PVA/CS-Ag films were close to those determined by AAS and indicate the presence of silver nanoparticles within the film network. The SEM pictures are in agreement with the obtained results from the FTIR/ATR spectra, where there is a defined intermolecular interaction between PVA and CS.

#### 3.3.3. Swelling Behavior

The swelling capacity of an ideal antibacterial composite film plays an essential role in its application, its ability to swell allowing the release of silver ions and drug.

**Figure 6:**
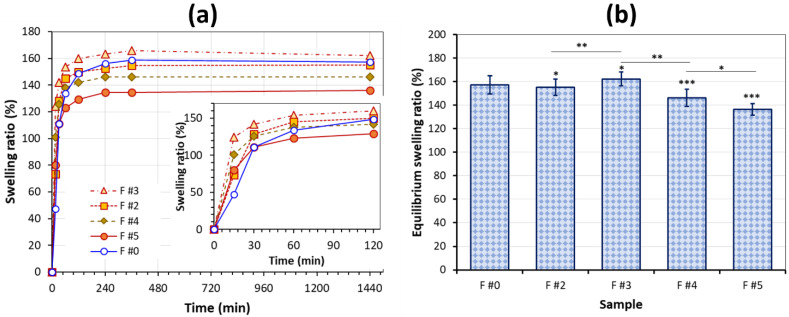
Swelling kinetics (**a**) and swelling degree at equilibrium (72 h) (**b**) of PVA/OxCS and composite PVA/OxCS-Ag films measured in phosphate buffer pH 6.8 and 37 °C. Results are expressed as mean ± standard deviation (*n* = 3). * *p* < 0.05, ** *p* < 0.01, *** *p* < 0.001, compared with F #0.

The literature data are controversial: while some researchers state that increasing the silver content in the films decreases or even does not change the hydrophilicity [55,92], on the contrary, others note an increase in the hydrophilicity [83,93]. The impact of the incorporation of silver nanoparticles on the PVA/OxCS film’s swelling behavior in time was evaluated. As shown in Figure 6a, for all films, the water absorption rate increased rapidly in the first 30 min and then slowly until the equilibrium state was reached, indicating a great hydrophilicity and flexibility of the cross-linked network. The incorporation of AgNps from 1.07 to 3.15 wt.% improved the swelling capacity of the films, while beyond 3.15 wt.%, a fall in water sorption capacity is distinguished. The maximum swelling ratios were 157, 155, 162, 146, and 136% for F #0, F #2, F #3, F #4, and F #5 films, respectively (Figure 6b). This behavior could be caused either by stretching of the polymer matrix due to the presence and interactions of AgNps with polymer chains or due to the solvation shell formed by water molecules bonded to AgNps [94]. However, samples F #4 and F #5 with higher nanoparticle content (>3.15 wt.%) showed a reduced swelling capacity (146% and 136%, respectively). In fact, additional H-bondings between the CS covering AgNps and hydroxyl and amine groups present in PVA/chitosan chains restrict the penetration of water and reduce overall swelling behavior (Figure 6b) [95,96]. Moreover, a greater number of silver nanoparticles occupy a larger space in the pores of material, which obviously results in a low swelling ratio of the composite film. It must also be annotated that all composite PVA/OxCS-Ag films were stable during 72 h of the swelling experiment, proving their excellent integrity and suitability for the intended application.

#### 3.3.4. Mechanical Properties

**Figure 7:**
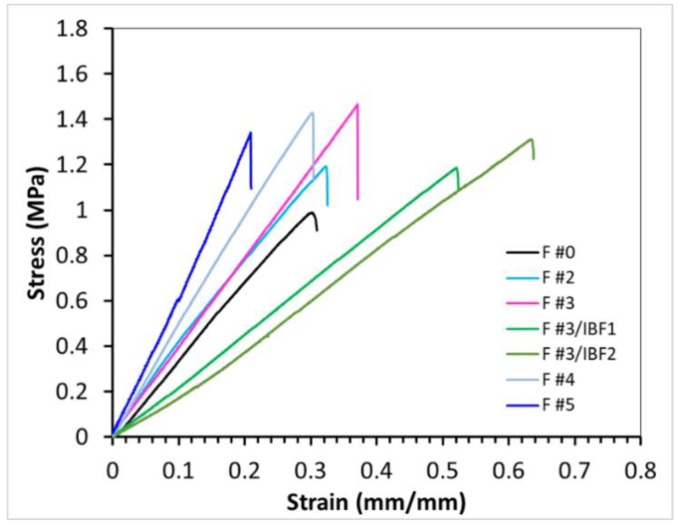
Stress−strain curves of PVA/OxCS and composite PVA/OxCS-Ag films without and with ibuprofen.

The mechanical strength of the composite films is a crucial factor in determining their ability to maintain integrity in the environmental conditions. The stress–strain curves are shown in Figure 7, and the values for the tensile strength, tensile modulus, and elongation at breakage of the films are presented in Table 2. An increase in the mechanical stability, such as tensile strength (TS) and elongation to break (EB), of the AgNps-loaded films was observed with increasing AgNps content up to 3.15 wt.% compared with pure PVA/OxCS film. The TS of F #0 film without AgNps is 0.97 ± 0.07 MPa and % of EB is 29.27 ± 1.37 (Table 2). By adding AgNps, both TS and EB (%) increase with the highest values for the F #3 sample (*p* < 0.001). A further increase in AgNps amount determined a slight decrease in TS and EB in the F #4 and F #5 samples. The surface of AgNps was stabilized by hydrophilic CS, and as long as the AgNps content in polymer matrix increases, the number of functional groups that can be involved in intermolecular interactions or even additional cross-linking (i.e., acetal bonds) increases (see Table 1). Consequently, the flexibility of the films decreases, making them stiffer. The results are in good agreement with those obtained for Young’s modulus (Figure 7). Therefore, an increase of 2.4-fold of Young’s modulus value (*p* < 0.001) (from 2.63 ± 0.5 MPa for F #0 to 6.3 ± 0.35 MPa for F #5) by adding 6.11 wt.% AgNps compared with the film without metallic nanoparticles was observed; this finding was in agreement with other studies [11,97]. In order to handle the film properly, the film is expected to be tough and hard without being brittle; consequently, we chose the F #3 sample as the best candidate for the next studies.

#### 3.3.5. Bioadhesive Properties

For local buccal administration, the polymeric film must adhere to the mucosa, and it is essential to have an adequate degree of mucoadhesion. The film without AgNps showed weaker adhesion properties than the composite PVA-OxCS-Ag films (Table 2). This behavior is related with the hydrophilicity (swelling degree) of respective films (see Figure 6). Usually, the introduction of silver nanoparticles leads to a reduction in the adhesion properties due to increased hydrophobicity [98]. In our case, the increase in CS content [99] in F #4 (*p* < 0.001) and F #5 (*p* < 0.001) brings more hydrophilic groups, which can bind with the mucin through hydrogen bonding or electrostatic interactions (with sialic acid) and facilitate muchoadhesion [100]. The results are comparable with those found for gellan gum films [100] or pectin films [101] and are within the allowed required range for the adhesion process (0.3–1.3 N) [102].

### 3.4. Preparation and Characterization of IBF-Loaded PVA/OxCS-Ag Films

Due to the swelling and mechanical performances, the F #3 sample was selected for further loading and release experiments. An increase in the drug content in the film by increasing the concentration of aqueous solution of IBF from 1 to 5 mg/mL was observed. As follows, the F #3 sample with low (61.7 mg/g (F #3/IBF1)) and high loading capacity (117.0 mg/g (F #3/IBF2)) was characterized for further use as potential drug delivery system in periodontitis treatment.

**Figure 8:**
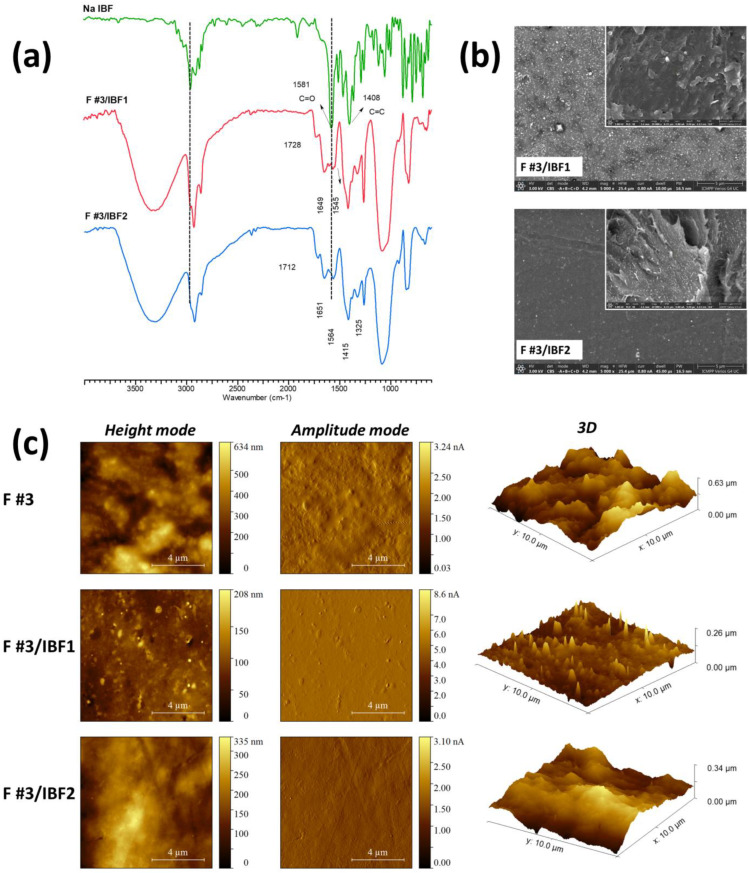
FTIR/ATR spectra (**a**), surface and cross-section morphology obtained by SEM analysis (**b**), and surface AFM images (**c**) of the unloaded and ibuprofen-loaded PVA/OxCS-Ag films.

The FT-IR/ATR spectra of the IBF-loaded PVA/OxCS-Ag films (Figure 8a) show new molecular features of the initial PVA/OxCS-Ag spectrum, such as 3308 (OH/NH_2_), 1720 (C=O acetate band in PVA), 1652 (amide I), and 1088 (C-O) cm^−1,^ as well as 2957, 1564, 1290, and 789 cm^−1^ from ibuprofen. However, the shift of the carbonyl (C=O) stretching band at 1585 cm^−1^ of sodium ibuprofen to a lower value of 1562 cm^−1,^ and of the aromatic ring (C=C) vibration band from 1408 cm^−1^ to 1415 cm^−1^ [103,104], highlighted a complete complexation between the free NH_2_ groups of OxCS and the carboxyl groups of IBF in both samples. An enhancement of the band corresponding to the stretching vibration of the ionized groups at 1560 cm^−1^ and the absence of the band at 1600 cm^−1^ from free NH_2_ groups in the spectrum of the F #3/IBF2 sample are also observed as a result of complexation with the drug. The changes can be attributed to the transformation of the sodium carboxylate ion pair into an ammonium carboxylate ion pair [105].

A small difference in the morphology of IBF-loaded films, both on the surface and in the section, was observed by SEM analysis due to the presence of aggregates resulting from the formation of complexes between CS and IBF (Figure 8b). Obviously, a higher amount of IBF in the film (F #3/IBF2 sample) has a greater covering and smoothing effect of the surface owing to the almost total complexation of the -NH_2_ groups of CS with COOH groups of IBF, as previously demonstrated by FT-IR analysis.

The morphology of the films was also visualized by the AFM technique in the amplitude and height profile, as well as 3D. Tapping-mode AFM height images (Figure 8c) show the presence of spherical AgNps distributed throughout the matrix of the F #3 sample with some agglomerations in the form of bunches, probably formed during the drying stage. The corresponding 3D image reveals a hill–valley-structured surface in the case of the sample without IBF with a RMS (Root Mean Square) roughness around 105 nm. When IBF was added, the AFM images showed distinct aspects of the surface roughness, probably due to the different IBF amount in the composite films. At a low IBF concentration, a slightly different sharp aspect in the 3D image is observed. The RMS roughness of the scanned surface is around 20 nm (F #3/IBF1 sample). Increasing the amount of IBF (F #3/IBF2 sample), the surface became smoother (more distinct in the phase image) and the film morphology appeared more homogenous, probably due to the smoothing effect of IBF. In this case, the 3D image shows mixed roughness, but the RMS roughness measured is around 50 nm.

**Figure 9:**
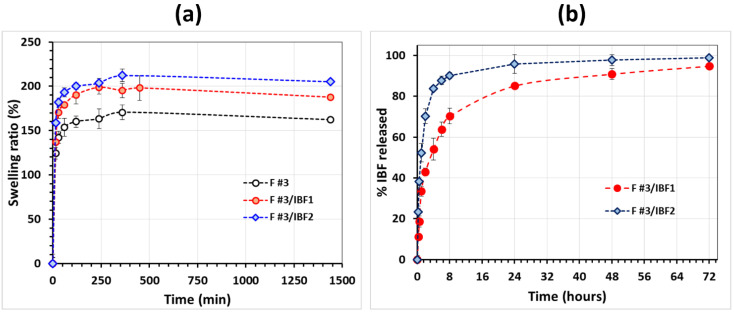
Swelling ratio (**a**) and release profiles of ibuprofen (**b**) of/from composite PVA/OxCS-Ag films with two different drug loading capacities in phosphate buffer at pH 6.8 and 37 °C. Results are expressed as mean ± standard deviations (n = 3).

The swelling capacity of the composite films with IBF was studied in a simulated physiological environment (phosphate buffer with pH 6.8) taking into account the salivary pH in conditions of periodontal disease (Figure 9). The water absorption capacity increased with the increase in the drug content. This fact is related to the free amino groups of CS which are available in larger quantities in the F #3/IBF1 (63%) than in F #3/IBF2 sample (37%). However, the swelling process is relatively fast, the equilibrium being reached after 4 h, caused by the partial release of the drug during the swelling experiment. This remark is also sustained by the weaker mechanical properties (tensile strength, elongation at break, and elastic modulus) of the composite films loaded with IBF (Figure 7, Table 2). In fact, following the involvement of amino groups in electrostatic bonds with the drug, the number of hydrogen bonds between polymers is reduced, and therefore the film becomes more flexible. This capacity of drug-loaded composite films to swell could also be responsible for the higher mucoadhesive force [106,107]. An opposite trend was reported by Ahmed and co-workers [17] for tetracycline-loaded chitosan films cross-linked with glutaraldehyde. An increase in tensile strength with an increase in drug content was observed, showing a minimum value for drug-free films. Conversely, the elongation percentage decreased with increasing both the drug amount and the cross-linking reaction time.

In vitro release of ibuprofen from PVA/OxCS-Ag film was carried out under fully immersion conditions, using a static model at pH 6.8 and 37 °C (Figure 9b). Both samples presented a quick release, however, the released amount of IBF from the sample with a higher loading capacity was larger in the first hour (52.35 ± 1.9% from F #3/IBF2 and 42.9 ± 2.7% for F #3/IBF1). The burst effect is probably due to the elution of the ibuprofen physically entrapped on the outer surface and cut edges of the film matrix. Then, in the next 7 h, almost the same difference is kept, after which the curves start to flatten. The entire amount of loaded IBF was released from both samples in 72 h. A slower release rate of IBF (up to 80% in 72 h) was obtained for PVA/CS/AgNps films cross-linked with glutaraldehyde and MgSO_4_ [55].

### 3.5. Antibacterial Properties

**Figure 10:**
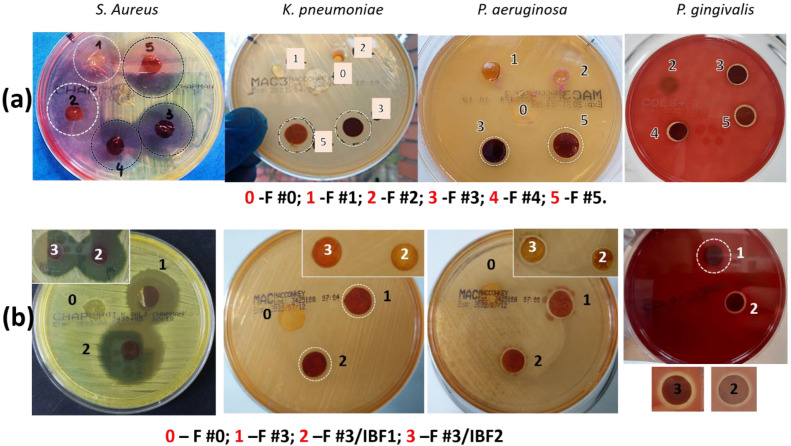
Comparative studies of antibacterial activity of pure and composite PVA/OxCS-Ag films (**a**) and of unloaded and ibuprofen-loaded composite films (**b**) against *S. aureus*, *K. pneumoniae*, *P. aeruginosa*, and *P. gingivalis* bacteria.

The antibacterial action of synthesized unloaded and IBF-loaded composite PVA/OxCS-Ag films is an important factor for determining their medical applications. For testing the antibacterial activity, equally weighed film samples containing different amounts of Ag (F #1 ÷ F #5 samples) and F #3 samples loaded with 6.17% (F #3/IBF1) and 11.7% IBF (F #3/IBF2) were subjected to disk diffusion method. After 24 h of incubation at 37 °C, the inhibition zone was observed in each of the samples (Figure 10, Table 3). The results shown in Figure 10a indicate a broad activity against pathogenic organisms such as *S. aureus*, *K. pneumoniae*, *P. aeruginosa*, and *P. gingivalis*, as well as the increase in the zone of inhibition with increasing amount of silver in the composite films, as shown in Table 3. However, a superior activity toward Gram-positive bacteria than Gram-negative was observed, which could be related to the attachment of Ag nanoparticles to the cell wall [108].

The Ibuprofen-loaded composite films had a higher antibacterial activity than free films, being more effective against *S. aureus* than *P. aeruginosa*, *K. pneumoniae*, and *P. gingivalis*, as indicated by the bigger inhibition zone (Figure 10b). It is known that ibuprofen is an antibacterial agent only for Gram-positive species, with little effect on Gram-negative ones [109]. The antibacterial effect can be related with the released amount of the ibuprofen from composite films, the diameter of the inhibition zones being larger for composite films with a higher amount of ibuprofen (F #3/IBF2 sample). The antibacterial activity of ibuprofen-loaded PVA/OxCS-Ag films demonstrated that they could not only achieve local and systemic drug delivery but also prevent oral bacterial infection.

### 3.6. Film Biocompatibility

**Figure 11:**
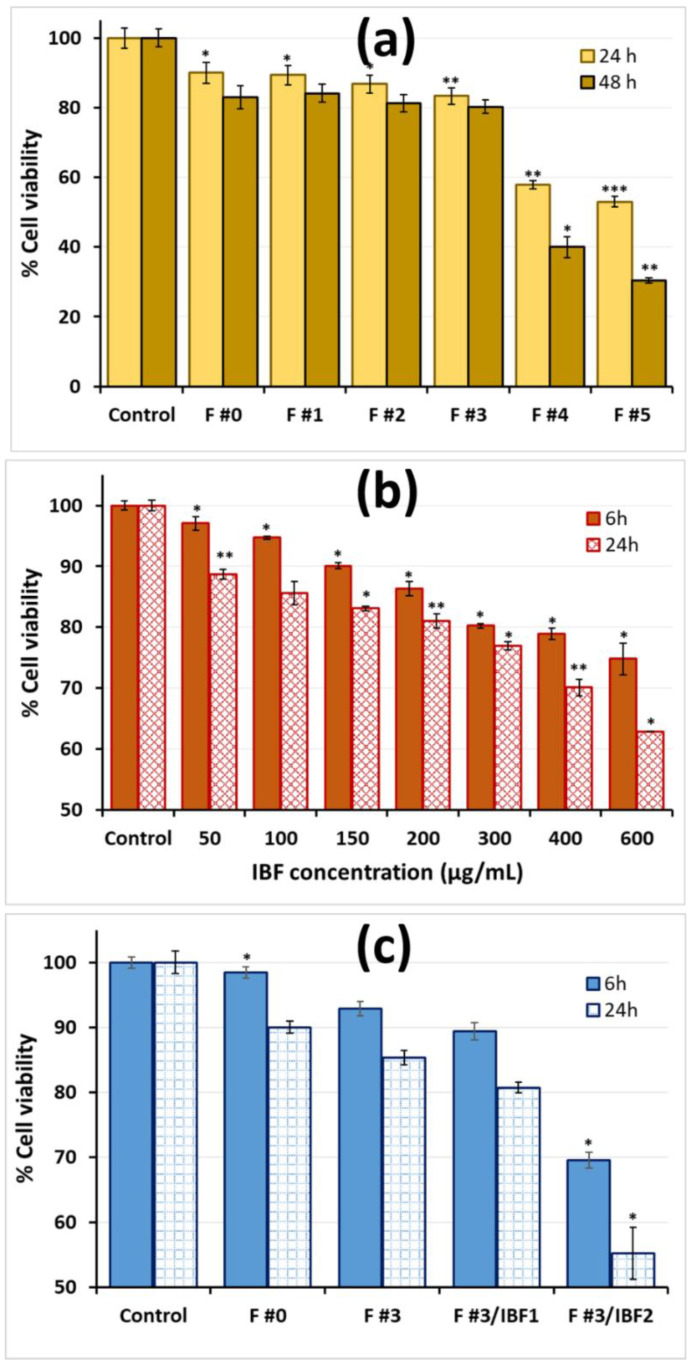
Viability of human dermal fibroblast adult (HDFa) cells exposed for different periods of time to pure and composite PVA/OxCS with AgNps (**a**), different sodium ibuprofen concentrations (**b**), and composite PVA/OxCS-AgNps with different ibuprofen content (**c**), assessed by MTT assay. Results are expressed as mean ± standard deviations (n = 3). * *p* < 0.05, ** *p* < 0.01, *** *p* < 0.001, compared with control (untreated cells).

The potential application of composites containing metal nanoparticles in the medical field demands their biosafety assessment. Furthermore, the cytocompatibility of pure and composite PVA/OxCS-Ag films, unloaded or loaded with IBF, was evaluated through in vitro studies. First, the human adult fibroblast (HDFa) cell’s viability was assessed after exposure to composite films with different AgNps content for 24 and 48 h. MTT assay results (Figure 11a) show a decrease in cell viability in response to the increase in AgNps content with more than 80% survival for the films with less than 1.44 wt.% Ag (F #1 ÷ F #3 samples). At higher Ag contents, cell viability drastically decreased, with the toxicity increasing more after 48 h of exposure.

When choosing a material for bioapplications, it is crucial to be safe regarding the concentrations of active components. The literature established that AgNps accumulate in brain cells in a time- and dose-dependent manner [110,111] and induce in vivo repeated-dose toxicity in mice [112]. Therefore, it is best to decide on the lowest possible AgNps concentration which provides suitable antibacterial effect. Since the F #3 sample containing 1.44 wt.% Ag presented both good antibacterial activity (Figure 10 and Table 3) and no cytotoxicity (Figure 11a), it was chosen as the best option for drug loading (see Section 3.5).

The next step was to determine which concentration of IBF would be non-cytotoxic for cells. So, different concentrations of sodium ibuprofen were tested for their effect on viability of fibroblast cells (Figure 11b). The results show that HDFa cells tolerated well (>80% cell viability) concentrations of IBF up to 300 µg/mL and 200 µg/mL after 6 h and 24 h of exposure, respectively. The results are consistent with those found by Canton et al. [113], who found a toxicity of IBF at concentrations higher than 10^−3^ M.

Finally, the pure PVA/OxCS film (F #0), the composite F #3 sample unloaded and loaded with two different amounts of IBF (F #3/IBF1 and F #3/IBF2), was placed in contact with HDFa cells for the same duration as for a simple drug (Figure 11c). The highest cytotoxic effect was shown by the film with higher drug loading capacity (F #3/IBF2). Even after 6 h of exposure, the cell viability was under 70%.

The release of ibuprofen from the F #3/IBF1 film with 6.17 wt.% IBF does not seem to affect cell viability to the same extent as free IBF. The concentrations of IBF released from the film disk (~2 mg) in 0.3 mL media at 6 h and, respectively, 24 h (considering that approximately 63.8% and 85.09% of IBF were released from the film at these times (Figure 9b)) are 262 µg/mL and 567 µg/mL. Cell viability increased from 83% to 89.4% at 6 h and from less than 70% to 80.7% after 24 h for free IBF and loaded film with IBF, respectively.

Based on the above-mentioned observations, we concluded that these films are the best option for periodontal treatment.

### 3.7. Performances and Limits of the Current Studies

Currently, there are not sufficient data to differentiate one particular local drug delivery system as superior over the others. Though some local drug delivery systems have been approved by FDA for the management of periodontal disease, the risk/benefit ratio concerning the usage of these drugs has yet to be established. Furthermore, continuous research in this field ensures and enables the usage.

Most of the film formulations proposed as local drug delivery systems for periodontal intrapocket treatment use glutaraldehyde (toxic reagent) as a cross-linker (Table 4) to improve the stability of the matrix network and then to achieve a sustained and suitable drug release rate. To the best of our knowledge, there is no other research reporting the use of oxidized chitosan both as matrix component and biodegradable cross-linker for the synthesis of composite PVA/CS films with AgNps. Compared with the performances of other films based on CS and its blends (Table 4), the synthesized film based on ibuprofen-loaded PVA/OxCS-Ag reveals good physico-chemical and biological properties, achieving both systemic drug delivery and preventing oral bacterial infection. However, despite the many advantages, practical applications require some improvements in the film properties. Thus, films do not always have the mechanical strength necessary to withstand prolonged mastication. Moreover, drug loading capacity is limited due to the small area of the film; moreover, a long-step preparation procedure as well as manufacturing cost should be taken in consideration. The in vitro release of IBF is sustained for up to 72 h. Additional studies must be performed to demonstrate the synergistic effect of the two drug components (AgNps and IBF) toward wound healing in the periodontal intrapocket.

## 4. Conclusions

In this study, PVA/CS composite films containing silver nanoparticles and ibuprofen were prepared and evaluated for their morphology, mechanical properties, loading content, ibuprofen release, and biological activity. The results can be summarized as follows:

(i) Sustainable, solvent-free, and green approaches were used to develop both the AgNps and IBF-loaded PVA/CS composite films. It was demonstrated that oxidized CS can be successfully used both as cross-linker and matrix component.
(ii) Small spherical AgNps (~10 nm) covered and stabilized by CS were obtained by optimizing synthesis parameters (5 mg/mL CS solution, 70 mM AgNO_3_, 90 °C, and 17 h). Chitosan assured the compatibility between the metal and the matrix in the final composite material.
(iii) The swelling capacity and morphological and mechanical properties of the composite films are influenced by the PVA/CS ratio, as well as by the amount of entrapped AgNps.
(iv) The composite film with 61.7 mg/g IBF showed a sustained release rate during 72 h in simulated artificial fluid.
(v) IBF-loaded composite PVA/OxCS-Ag films displayed a greater inhibitory effect than unloaded PVA/OxCS-Ag films (better antimicrobial activity on *S. aureus* than *P. aeruginosa*, *K. pneumoniae*, and *P. gingivalis*) and no cytotoxicity to HDFa cells.
(vi) Finally, film formulation with 1.44 wt.% Ag and 61.7 mg/g IBF has good bioadhesive properties and no cytotoxicity, which recommend it for use in periodontal treatment.

## Figures and Tables

**Table 1:** Chemical composition of the initial mixture and of PVA/OxCS films without and with AgNps.

Sample Code	Initial Mixture Composition				Final Film Composition (%, *w*/*w*) **			
	PVA (%, *w*/*w*)	OxCS (%, *w*/*w*)	CS-AgNps Solution (mL)	CS-AgNps (%, *w*/*w*) *	PVA	CS	Ag	
							Theoretic	Determined by AAS ***
**F #0**	66.66	33.33	-	-	77.3 ± 0.12	22.7 ± 0.12	-	-
**F #1**			0.75	1.07	80.77 ± 0.74	18.68 ± 0.86	0.42	0.55 ± 0.063
**F #2**			1.5	2.12	79.36 ± 0.48	19.61 ± 0.56	0.82	1.03 ± 0.069
**F #3**			2.25	3.15	75.33 ± 0.25	23.23 ± 0.28	1.22	1.44 ± 0.105
**F #4**			2.75	4.16	73.13 ± 0.47	24.58 ± 0.51	1.61	2.29 ± 0.213
**F #5**			3.5	6.11	64.54 ± 0.88	32.28 ± 1.02	2.36	3.18 ± 0.118

* Relative to the polymer mixture; ** Determined by ninhydrin assay [67]; *** AAS-atomic absorption spectroscopy.

**Table 2:** Mechanical properties of pure, unloaded and ibuprofen-loaded composite PVA/OxCS-Ag films.

Sample Code	Tensile Strength (MPa)	Young’s Modulus (MPa)	Elongation to Break (%)	Adhesion Force (N)
**F #0**	0.97 ± 0.07	3.42 ± 0.36	29.27 ± 1.37	0.26 ± 0.026
**F #2**	1.19 ± 0.05 *	3.86 ± 0.41	32.63 ± 1.70	0.41 ± 0.031 **
**F #3**	1.46 ± 0.06 ***	3.92 ± 0.11	37.18 ± 3.60 *	0.50 ± 0.050 **
**F #4**	1.42 ± 0.09 **	4.76 ± 0.47 *	30.30 ± 7.00	0.56 ± 0.042 ***
**F #5**	1.32 ± 0.05 **	6.30 ± 0.32 ***	20.68 ± 2.02 **	0.60 ± 0.055 ***
**F #3/IBF1**	1.18 ± 0.08 *	2.31 ± 0.12 **	52.33 ± 4.11 ***	0.71 ± 0.030 ****
**F #3/IBF2**	1.10 ± 0.01 *	2.14 ± 0.10 **	63.75 ± 2.65 ****	0.90 ± 0.025 ****

Results are expressed as mean ± standard deviations (n = 3). * *p* < 0.05, ** *p* < 0.01, *** *p* < 0.001, **** *p* < 0.0001, compared with control (F #0 sample).

**Table 3:** Inhibition zone diameters of the composite PVA/OxCS-Ag and ibuprofen-loaded PVA/OxCS-Ag films against different microorganisms.

Sample	Zone of Inhibition Diameter (mm)			
	*Staphylococcus aureus*	*Klebsiella pneumoniae*	*Pseudomonas aeruginosa*	*Porphyromonas gingivalis*
**F #0**	- *	-	-	-
**F #1**	15.8 ± 0.32	-	-	-
**F #2**	16.8 ± 1.03	-	-	-
**F #3**	21.2 ± 0.75	1.6 ± 0.02	1.5 ± 0.14	1.7 ± 0.11
**F #4**	22.4 ± 0.36	n.d. **	n.d.	2.0 ± 0.10
**F #5**	23.4 ± 0.72	3.85 ± 0.13	1.8 ± 0.24	2.3 ± 0.14
**F #3/IBF1**	21.5 ± 0.65	1.69 ± 0.02	1.7 ± 0.12	2.1 ± 0.07
**F #3/IBF2**	22.6 ± 0.40	2.86 ± 0.10	2.38 ± 0.11	3.17 ± 0.09

* no activity; ** not determined.

**Table 4:** Film as intrapocket drug delivery system for the treatment of periodontitis.

Polymer	Cross- Linker	Drug/Inorganic Particles	Performances	Ref.
CS	TPP	Taurine/-	Taurine incorporated in chitosan film seems to enhance the effect of chitosan on tissue repair in periodontitis	[40]
CS	TPP	Chlorhexidine Gluconate/-	Enhance antimicrobial activity against *P. gingivalis*	[13]
CS	GA	Ciprofloxacin Diclofenac/-	Good tensile strength; burst release of 40% drug during first 24h, followed by a slow release up to 21 days; inhibitory effect on *S. mutans* up to 24 days	[18]
CS	GA	Lyncomycin/ -	Good folding endurance; sustained release without any burst effect for consecutive 5 days	[19]
CS	GA	Metronidazole Levofloxacin/ -	Good antibacterial activity against *S. aureus* and *E. coli*; after treatment of 8 weeks: pale pink gum, no signs of swelling or inflammation along with swift reattachment of periodontal ligament and significant reduction in pocket depth	[20]
CS	GA	Risedronate/ Zinc HAP	Effectively reduced alveolar bone destruction and contributes to periodontal healing in a rat model of experimental periodontitis	[21]
CS	GA	Metformin/ -	Burst release of 75% drug during first 24h, followed by a slow release up to 11 days; good antibacterial activity and improved alveolar bone properties in a rat model	[22]
CS	-	Sparfloxacin/ -	Tensile strength of the film increased with the increase in adjuvants; antimicrobial activity was retained for 96 h	[23]
CS, PVA	GA	-/bioactive glass	Better mechanical properties for films with 0–17% glass; in vitro bioactivity increases with glass content in the hybrid	[42]
CS, PVA	-	Meloxicam/ HAP	fibers showed comparatively sustained release of the drug over a 24 h period; fibers gave the maximum cell viability and growth while films’ formulation did not support the cells’ growth and proliferation	[114]
CS, PVA	-	Astaxanthin/graphene oxide	Good tensile strength; antibacterial performance, a very good inhibitory effect on *E. coli*; good cell compatibility	[115]
CS, PVA	Genipin	Tetracycline/-	Sustained release of drug over 14 days; cytocompatibility; only structures containing tetracycline exhibited expressive antibacterial activity against periodontal pathogens	[43]
PVA, CMCS	-	Ornidazole/-	Slow drug release rate enhanced by increasing the poly(vinylalcohol) in the system; excellent biological properties, no hemolysis and no toxicity to rat periodontium	[116]
CS, CMCS, PVA	GA, TPP	-/-	No hemolysis, low protein adsorption; favorable degradability; chitosan microspheres incorporated in thin poly(vinylalcohol) films could be easily inserted in periodontal pocket; microspheres provide sustained drug release	[35]
CS, PLGA	-	Ipriflavone/-	Release of Ipriflavone for 20 days from monolayer film	[28]
CS, P(e-CL)	-	Metronidazole benzoate/-	mucoadhesive film with acceptable drug release and mechanical and bioadhesion properties; local delivery of the drug in a concentration higher than the metronidazole minimum inhibitory concentration against anaerobic bacteria	[24,25]
CS, PVP	-	Tenoxicam/-	Adequate mechanical and bioadhesion properties for buccal films; rapid and sustained salivary TNX delivery in the buccal cavity	[26]
CS, Alginate	CaCl_2_	Clindamycin phosphate/-	Higher adhesiveness and cohesive and flexible film; higher swelling ability; delayed drug release until 10h	[41]

CS—chitosan; PVA—poly(vinyl alcohol); PVP—poly(vinyl pyrrolidone); P(e-CL)—poly(epsilon-caprolactone); PLGA—poly(lactic-co-glycolic) acid; TPP—tripolyphosphate; GA—glutaraldehyde.

## Data Availability

The data presented in this study are available on request from the corresponding author.
